# A Hierarchical Feature Extraction Network for Fast Scene Segmentation

**DOI:** 10.3390/s21227730

**Published:** 2021-11-20

**Authors:** Liu Miao, Yi Zhang

**Affiliations:** National Key Laboratory of Fundamental Science on Synthetic Vision, Sichuan University, Chengdu 610017, China; leomiao@stu.scu.edu.cn

**Keywords:** semantic segmentation, scene understanding, hierarchical feature extraction

## Abstract

Semantic segmentation is one of the most active research topics in computer vision with the goal to assign dense semantic labels for all pixels in a given image. In this paper, we introduce HFEN (Hierarchical Feature Extraction Network), a lightweight network to reach a balance between inference speed and segmentation accuracy. Our architecture is based on an encoder-decoder framework. The input images are down-sampled through an efficient encoder to extract multi-layer features. Then the extracted features are fused via a decoder, where the global contextual information and spatial information are aggregated for final segmentations with real-time performance. Extensive experiments have been conducted on two standard benchmarks, Cityscapes and Camvid, where our network achieved superior performance on NVIDIA 2080Ti.

## 1. Introduction

The task of semantic segmentation has a long history [1], including scene parsing and holistic understanding [2]. It has attracted more attention in recent years due to its applications in the field of environmental perception [3,4], autonomous driving [5,6,7,8,9], and virtual reality [10,11], etc. The development of deep convolutional neural networks (DCNN) has promoted remarkable progress on semantic segmentation, especially after the emergence of fully convolutional networks (FCN) [12]. Most existing methods utilize FCN to produce dense predictions by assigning class labels for every pixel in a given image, which cause heavy computational load in object classification stage. Therefore, reaching a balance between inference speed and accuracy is of vital importance and is also a challenging task.

Till now, many schemes have been published with satisfactory results (e.g., [12,13,14,15,16,17]) using deep CNN with encoder-decoder frameworks. Since global contextual information (the correlations among different classes) and spatial details (the fine-grained boundary information) are both crucial to the segmentation process. The encoder adopts a heavy backbone to extract rich global contextual information by down-sampling the input image [12]. While skip-connections [12,13] and atrous convolutions [14] are also presented to mitigate the loss of spatial details. Meanwhile, the decoder utilizes an up-sampling strategy to recover the spatial details [13] based on multi-layer features. Daquan Zhou et al. [15] made a comprehensive study on the structure and shortcomings of a previous inverted residual block, and rethought the necessity of design changes. They analyzed the performance of a sandglass block by connecting high-dimensional representations. However, the improvement in performance would be at the cost of increased computational complexity of the backbone (i.e., ResNet50, 101 [18]), and the inference time would be much longer. Under this situation, some fast segmentation algorithms (e.g., BiSeNet [19]) emerge to accelerate inference process, which employ 2-branch or multi-branch architectures. Normally, the deep branch extracts a global context and the shallow branch captures spatial detail. These architectures indeed have fewer operations, but the decoders lack the ability of feature aggregation and may easily lose the fine-grained spatial details around boundaries or small objects. To make a balance between accuracy and speed, Changqian Yu et al. [20] put forward an improved version of BiSeNet [19] (dubbed BiSeNet V2). The core components include Spatial Path and Context Path to cope with the loss of spatial information and shrinkage of receptive field, respectively. Meanwhile, the Feature Fusion Module (FFM) and Attention Refinement Module (ARM) are also utilized to improve accuracy with tolerable cost.

Partially motivated by the success of recent works, we present HFEN (Hierarchical Feature Extraction Network), a lightweight semantic segmentation network with an encoder-decoder framework and a multi-branch architecture. Our network is simple yet computationally efficient, being able to extract features effectively by the encoder with the aid of inverted residual modules. While a multi-branch decoder is capable of aggregating multi-layer features from the backbone through a feature pyramid. Finally, a pyramid pooling module is applied to further refine the segmentation results.

**Figure 1:**
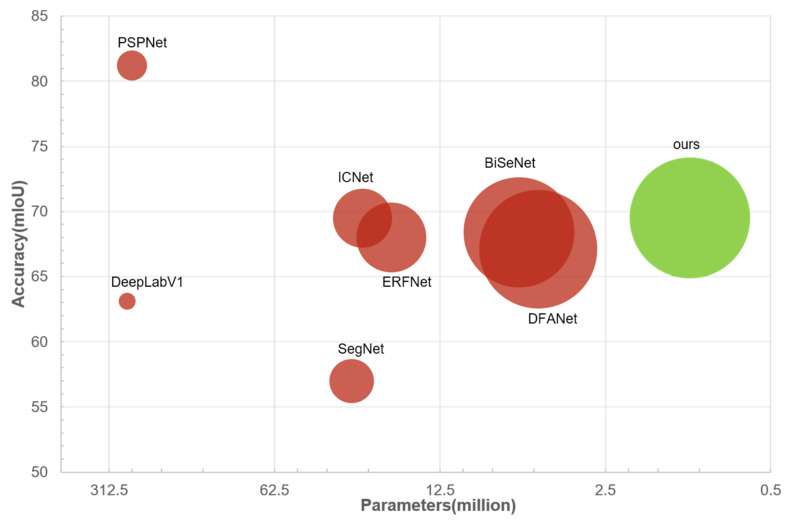
A comparison of the combined performance (including inference speed, accuracy, and number of parameters) among different methods. The bigger the circle, the faster the speed.

Our model has only 1.1 million parameters, which is relatively small under heavy architectures. In comparison, DeepLab_v1 [21] has 262.1 million parameters, PSPNet [16] has 250.8 million, and it is even fewer than some real-time networks (e.g., SegNet [17]: 29.5 M, ICNet [22]: 6.68 M, BiSeNet [19]: 5.8 M]). Nevertheless, we achieve the best overall general performance on Cityscapes [23] (inference speed vs accuracy) compared with existing methods (e.g., ICNet [22], SegNet [17], DFANet [24], BiSeNet [19], DeepLab [21], and PSPNet [16]), as shown in Figure 1 below:

Our major contributions are summarized as three-fold:

(1) We propose a lightweight architecture with an encoder-decoder framework to extract hierarchical features from the input image;
(2) An efficient encoder is proposed with inverted residual bottleneck modules to extracts global context information. A decoder is developed to aggregate multi-level hierarchical features, which effectively recovers spatial characteristics;
(3) Experiments have been conducted on the Cityscapes [23] and Camvid [25] dataset, where our network achieves a competitive result of 69.5% class mIoU and 87% category mIoU on Cityscapes and 66.1% mIoU on Camvid, while maintaining a real-time inference speed with a low memory footprint.

The rest of this paper is organized as follows: A brief overview of related works is placed in Section 2. The architecture of our network is described in detail in Section 3. Experimental results are exhibited in Section 4 with thorough analysis. A conclusion is drawn in Section 5.

## 2. Related Works

A solid foundation has been laid by [12] using fully convolutional networks (FCN) for image segmentation. Since then, more FCN-based work has sprung up and gained popularity. Under a general encoder-decoder framework, the encoder is employed to extract a global context [12], and a decoder is utilized to recover the spatial details through up-sampling operations. Considering higher-level features in a receptive field are critical for the extraction of a global context. FCN adopts VGG [26] as its encoder, then the decoder uses a skip connection from lower layers to recover the spatial details. U-Net [13] leverages dense skip connections to further analyze lower-level features. Atrous convolution is elaborated by DeepLab [21], in which a dilated kernel is used to expand the receptive field and maintain the resolution of features. PSPNet [16] later extends this idea by using dilated ResNet for feature extraction. However, the computational complexity increases significantly due to the high dimensions of features.

The pyramid pooling and atrous spatial pyramid pooling (ASPP) are developed by PSPNet [16] and Deeplab [21], respectively to further exploit the global context. The pyramid pooling module integrates the spatial features under four different scales. ASPP filters a convolutional feature layer with different sampling rates to delineate the objects. HRNet [27] is originally designed for human pose estimation, which starts from a high-resolution sub-network, and adds high-to-low resolution sub-networks step by step, then connects the mutli-resolution sub-networks in parallel. Such a scheme could also be used in the prediction of dense labels, which is later proved to have a good performance on the Cityscapes dataset. However, due to its deep multiple branches and duplicated fusion of multi-scale feature, HRNet fails to meet real-time demand.

The above-mentioned methods mainly focus on segmentation accuracy, sacrificing inference speed. In view of this, an astonishing amount of research efforts have been made to cater for the ever-growing demand for fast processing, in which an efficient framework is required to predict pixel-wise label in a real-time manner. Therefore, a small sized network with less computation is needed. SegNet [17] is a novel deep fully convolutional neural network for semantic segmentation. The encoder resembles the 13 convolutional layers in VGG16 [26], and the decoder aims to map the low-resolution feature maps to full input resolution. Moreover, GUN [28] and BiSeNet [19] employ a 2-branch architecture, while ICNet [22] performs the image cascade through a multi-branch architecture, where a deep branch is used to extract a global context for low resolution features and a shallow branch is utilized to capture spatial details. Later, Fast-SCNN [29] combines the encoder-decoder framework with 2-branch architecture, and realizes a better performance on high resolution images using efficient computational embedded devices with low memory. Albeit the higher processing speed, their accuracies are not satisfactory.

Furthermore, MobileNet [30] proposes a depth-wise separable convolution (DSConv) to accelerate the inference speed, which decomposes a standard convolution into a depth-wise convolution and a point-wise convolution. This method largely reduces the computational cost and memory footprint of the network with a slight loss of accuracy. Xception [31] and MobileNetV2 [32] further investigate the efficient design of the DSConv. In particular, Xception introduces an inception module into CNN as an intermediate operation in-between traditional CNN and DSConv. MobileNetV2 comes up with an efficient inverted bottleneck residual block that serves as a feature extraction backbone and achieves higher accuracy than MobileNet. ContextNet [33] extends this module and explores enhanced modules based on factorized convolutions, network compression, and multi-scale feature representation to produce impressive segmentation results with low memory requirement.

## 3. Description of Algorithm

### 3.1. Problem Formulation

A traditional encoder-decoder architecture for semantic segmentation is formulated as follows:

Let $I\in \;R^{H\times W\times 3}$ be the input to an encoder E, and $F\;\in \;R^{\tilde{H}\times \tilde{W}\times \overset{ˇ}{C}}$ is the corresponding output. Let $Y\;\in \;{\left\{0,\;1,\;2,\;\ldots ,\;C\right\}}^{H\times W}$ be the labels of ground truth, where $\overset{ˇ}{C}$ and C are the number of output channels (also known as width of the network) and number of categories, respectively. Y is a one-hot indicator, written as: $Y\;\in \;{\left\{0,\;1\right\}}^{H\times W\times C}$. Since semantic segmentation is a pixel-wise prediction, F shall be up-sampled by decoder D to match the size of Y before calculating training loss, which is written as:

$$F=E\left(I\right)$$

(1) $$L\left(F,\;Y\right)=Loss\left(softmax\left(D\left(F\right)\right),\;Y\right)$$

here cross-entropy is used to measure the loss.

The comprehensive performance of a segmentation algorithm is determined by both accuracy and inference speed, which turn out to be contradictory in most of the cases. Our goal is to reach a balance between these two metrics. In a segmentation process, an encoder consists of many convolutional layers taking up most of the computation, thus the scaling of the encoder is crucial to the overall performance. A typical convolutional layer is written as: $Y_{i}=F_{i}\left(X_{i}\right)$, here $X_{i}$ is an input vector with a shape of $\langle H_{i},\;W_{i},C_{i}\rangle$, $F_{i}$ is a designed mapping function for different scenarios, and $Y_{i}$ is the output vector. Here $H_{i}$ and $W_{i}$ are the height and width of the input (also known as resolution), while $C_{i}$ is the width of the network. A ConvNet N can be written as a cascade operation:

(2) $$N=F_{L}⊙\ldots ⊙F_{2}⊙F_{1}\left(X_{i}\right)={⊙}_{j=1\ldots L}F_{j}\left(X_{\langle H_{i},\;W_{i},C_{i}\rangle }\right)$$

where L represents the depth of the network. Normally, the convolutional layers are divided into different stages, in which the layers in each stage share the same structure as [34] (except that the 1st layer performs a down-sampling operation).

Let $g_{i}$ be the computational load of a single convolutional layer, and the computation for the entire network G is determined by: $g_{i}$, L, $C_{i}$, and $\langle H_{i},\;W_{i}\rangle$. 

To reduce $g_{i}$, our encoder employs inverted bottleneck residual blocks with an efficient depth-wise separable convolutional layer (DSConv). The input tensor of a standard convolution is written as $X_{i}\;\langle H_{i},\;W_{i},C_{i}\rangle$, a convolutional kernel is expressed as $K\;\in \;R^{k\times k\times C_{i}\times C_{j}}$, with k as its kernel size, and the corresponding output is $X_{j}\langle H_{i},\;W_{i},C_{j}\rangle$. A typical convolutional layer has a computational cost as:

(3) $$H_{i}\times W_{i}\times C_{i}\times C_{j}\times k\times k.$$

By comparison, a DSConv consumes a much lower cost, shown as:

(4) $$H_{i}\times W_{i}\times C_{i}\times \left(k^{2}+C_{j}\right).$$

For a 3 × 3 kernel, the computational load of (4) is less than 10% of (3), sacrificing only small portions of accuracy [30].

Our encoder is designed with a small L, $C_{i}$ to reach an optimal trade-off between inference speed and segmentation accuracy. The effectiveness of our network has been testified in an ablation study in Section 4.5.

### 3.2. Network Architecture

**Figure 2:**
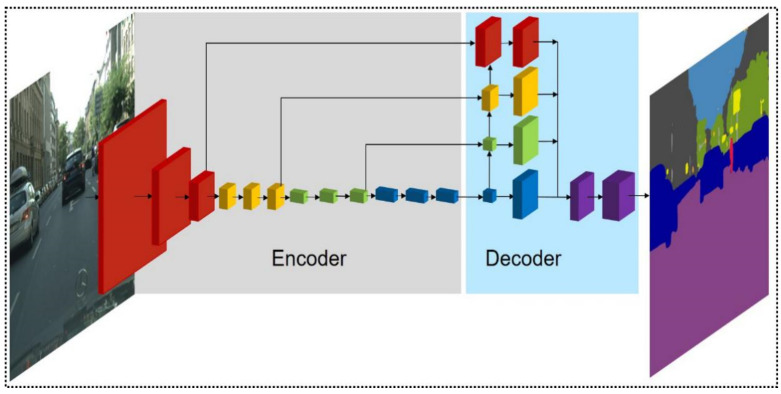
The architecture of Hierarchical Feature Extraction Network (HFEN). The size of each block represents the size of corresponding feature maps. While different colors show blocks at different stages in the encoder and their aggregated feature in the decoder.

To leverage the hierarchical semantic features from multiple levels, our network (dubbed HFEN) is constructed based on an encoder-decoder framework (shown in Figure 2 above), which consists of a top-down scaling, a bottom-up scaling, and an element-wise summation operation. Here “top-down” and “bottom-up” refer to 2 computation pathways in our hierarchical feature network, which involve down-sampling and up-sampling operations, respectively. Element-wise summation fuses the features from different levels to generate strong semantic features.

#### 3.2.1. Encoder

**Figure 3:**
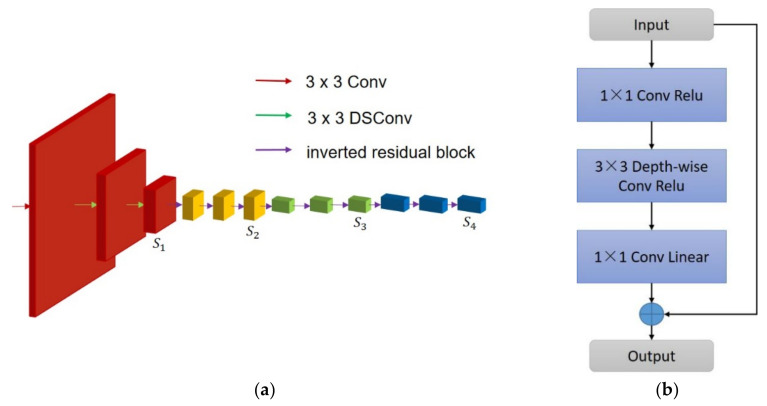
The structure of our encoder and inverted residual block. (**a**) Structure of encoder; (**b**) the processing flow of the inverted residual block.

Our encoder processes input images in a top-down manner (shown in Table 1 and Figure 3a below). The hierarchical features are computed at different scales with a scaling factor of 2. We stipulate those layers that produce the feature maps with the same size belonging to the same stage. Our encoder has 4 stages in total (marked red, yellow, green, and blue, respectively in Figure 2), with the output of each stage fed as input to the decoder.

To preserve the spatial details is crucial in the 1st stage. In this light, we place 3 layers to extract low-level features. The 1st layer is a standard 2D convolutional layer followed by 2 DSConv layers. Although DSConv is computationally more efficient, we insist on placing one convolutional layer in the 1st layer, since the input image has only 3 channels, hence the advantage of the DSConv convolutional layer could not be fully utilized. In the meantime, all layers adopt stride 2, followed by batch normalization (BN) [35] and ReLU, as well as a kernel size of 3 × 3.

Additionally, we insert efficient inverted residual blocks into the following 3 stages (labelled as purple arrows in Figure 3a). The structure of an inverted residual block is illustrated in Figure 3b, which consists of an efficient DSConv with small network scales. The linear bottleneck (1 × 1 Conv Linear) reduces the channel redundancy and computation load. In particular, the residual connection is added to learn the global context, when the input and output are of the same size.

The output of the 1st stage is directly fed to the 2nd stage (with 1/8 the resolution of the original input). Then the 3rd stage down-samples it again to 1/32 of the original size. The 4th stage maintains the resolution of the third stage, and only increases the number of channels. Finally, a hierarchical feature map is formed with the 4 stages. The outputs of the 4 stages are denoted as $\left\{S_{1},S_{2},\;S_{3},S_{4}\right\}$. And the strides for the input image at each stage are 8, 16, 32, and 32, respectively. A pseudo code for our encoder is written in Algorithm 1.
**Algorithm 1**: Encoder**Input**: RBG Image I 1: **I** is propagated through a Conv2D and two DSConvs →$S_{1}$; 2: **for** (i = 2; i <= 4; i++) 3: $S_{i-1}$ propagates through 3 Inverted Residual blocks respectively → $S_{i}$; **Output**$:\;\mathrm{Hierarchical}\;\mathrm{Feature}\;\mathrm{Set}\;\left\{S_{1},S_{2},\;S_{3},S_{4}\right\}$


#### 3.2.2. Decoder

Contrary to the encoder, our decoder is constructed in a bottom-up manner. As mentioned in the previous paragraph, the decoder takes the output of each stage of the encoder, and generates feature maps with proportional size at different levels, in a fully convolutional way.

The lower resolution features are processed in a bottom-up manner by up-sampling. These features are then merged with features from the encoder through an element-wise summation. Each summation fuses feature maps of the same spatial size via both bottom-up scaling and top-down scaling.

**Figure 4:**
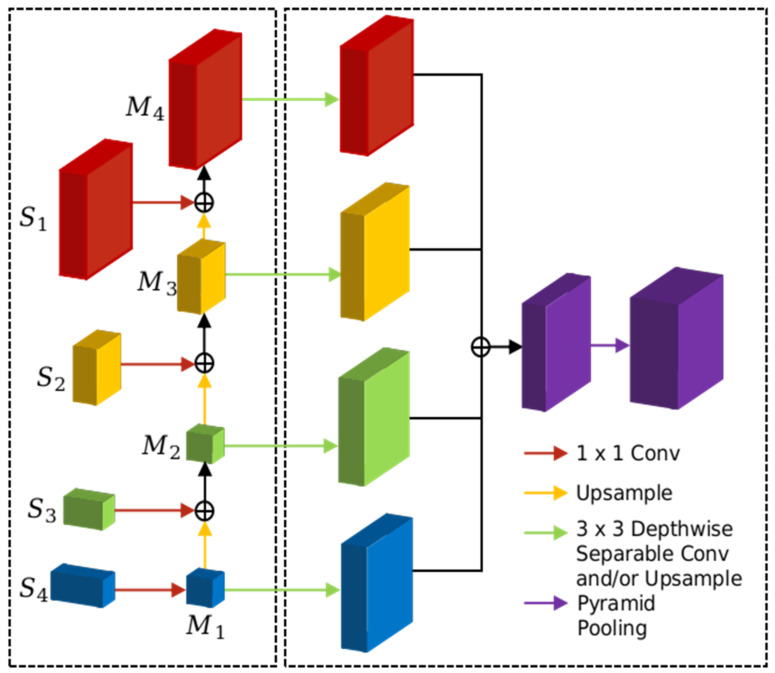
The structure of our decoder.

Figure 4 (left) shows the bottom-up structure of our decoder. The lower-resolution features are up-sampled by 2, and are then integrated with the corresponding features from the encoder via element-wise additions. Such an operation is iterated to create a map with the highest resolution. More specifically, we attach a 1 × 1 convolutional layer on $S_{4}$ to produce the lowest resolution map $M_{1}$, which is further up-sampled to the same size as $S_{3}$. While $S_{3}$ is also propagated through a 1 × 1 convolutional layer to maintain the same number of channels as $M_{1}$. An element-wise summation is performed for $M_{1}$ and $S_{3}$ to yield $M_{2}$. The above operation is iterated till we obtain a set of an integrated feature map $\left\{M_{1},M_{2},\;M_{3},M_{4}\right\}$.

To obtain the final segmentation output based on $\left\{M_{1},M_{2},\;M_{3},M_{4}\right\}$, a simple scheme is deployed to integrate the features from all levels (as shown in Figure 4 (right)). Considering that the strongest features are captured by the deepest layer, we hence start from the 1/32 ($M_{1}$) level, and perform 2 up-sampling operations to create a feature map at a 1/8 level. Each up-sampling operation consists of a 3 × 3 DSConv layer, a BN, a ReLU, and two bilinear up-samplings, each of which has a scaling factor of 2.

The above process is repeated for the next 3 levels (scales of 1/32, 1/16, and 1/8, respectively with fewer up-sampling at each level). The final maps contain a set of feature maps at a 1/8 scale, which are element-wise summed. A pyramid pooling module (PPM) [16] is appended to the end to aggregate the context information from different regions. The PPM is a 4-level module with output sizes of 1 × 1, 2 × 2, 3 × 3, and 6 × 6, respectively. The output of PPM is concatenated to the integrated feature maps from the last layer to generate the final feature representation. Finally, the representation is fed into a convolutional layer to obtain the final pixel-level segmentation results. A pseudo code for our decoder is written in Algorithm 2:
**Algorithm 2**: Decoder**Input**: Hierarchical Features$\left\{S_{1},S_{2},\;S_{3},S_{4}\right\}$ 1: $S_{4}$ propagates through a 1 × 1 Conv →$M_{1}$; 2: **for** (i = 3; i >=1; i−−) 3: $S_{i}$ propagates through a 1 × 1 Conv → $S_{i}^{\prime }$; 4: **while** (size of $S_{i}^{\prime }!$ = size of $M_{4-i}$) 5: perform bilinear up-sampling operation with a factor of 2 on $M_{4-i}$; 6: perform element-wise summation: $S_{i}^{\prime }$$M_{4-i}$ →$M_{4-i+1}$; 7: up-sample $\left\{M_{1},M_{2},\;M_{3},M_{4}\right\}$ to the same size then perform element-wise summation; 8: The combined features map propagates through PPM; 9: Perform up-sampling and Conv2D → final label map **Output**: Prediction Label Map

## 4. Experiment and Analysis

We implement our network on two standard benchmarks (Cityscapes [23] and Camvid [25]) to evaluate the general performance of our proposed network. A comparison is made among our method and other popular methods in terms of accuracy, network scale, and inference speed. Finally, an ablation study is carried out to verify the effectiveness of each module proposed in the overall architecture.

### 4.1. Datasets

#### 4.1.1. Cityscapes

Cityscapes is one of the most famous datasets of urban street scenes parsing. It contains 5000 fine annotated images, in which 2975 images are used for training, 500 images are used for validation, and 1425 images are reserved as testing samples. There are in total, 30 classes defined and we use 19 of them in our experiment. We obtained a 69.5% class level mIoU and 87% category level mIoU.

#### 4.1.2. Camvid

The Cambridge-driving Labeled Video Database (Camvid) is a dataset of road scenes taken by a moving vehicle. It consists of 701 finely annotated frames with a resolution of 960 × 720. It has 367 images for training, 101 images for validation, and 233 images for testing. The original annotation consists of 32 categories, but we merge them into 11 categories.

### 4.2. Implementation Details

Our training process is conducted on RTX 2080Ti GPUs with CUDA 11.1, CUDNN 8.0.5, and PyTorch 1.8.

#### 4.2.1. Cityscapes

We use the stochastic gradient decent (SGD) optimizer with a momentum of 0.9 and a weight decay of 0.00004. Inspired by [16], we set the initial learning rate as 0.08 and deploy the poly learning scheme with a power of 0.9.

**Figure 5:**
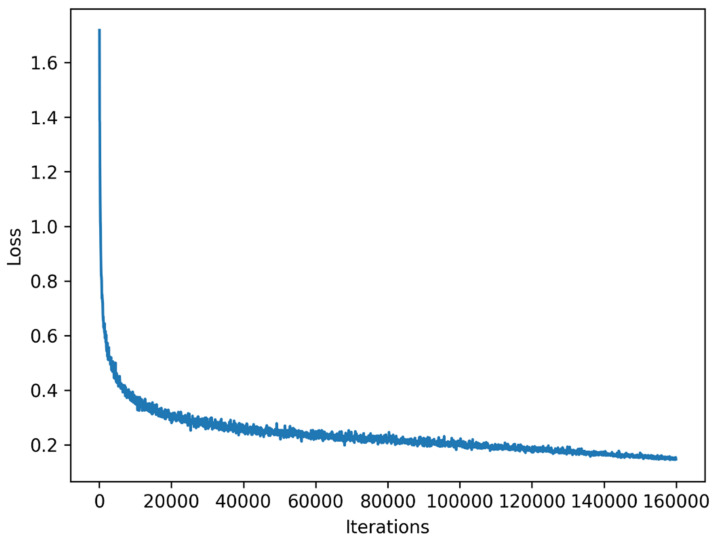
Loss over iterations on Cityscapes datasets.

We also adopt various data augmentation strategies to expand the training data, including random cropping images into 1024 × 512, random scaling in the range of 0.5 to 2.0, random horizontal flipping with probability of 0.5, and random photo metric distortion. Our model is trained with cross entropy loss for 160 k iterations (shown in Figure 5 below). The batch-size is set as 16, and synchronized batch normalization (SyncBN) [35] is deployed before non-linear functions.

#### 4.2.2. Camvid

Our configuration on the Cavmid dataset is similar, except that the crop size is set as 512 × 512 during data augmentation, with a batch-size of 44 and initial learning rate of 0.01.

### 4.3. Measurement

#### 4.3.1. Computational Complexity

The computational complexity of the trained models are measured by three metrics:

(1) Giga floating-point operations per second (GFLOPs):
  GFLOPs indicates the number of multiply-add operations required to evaluate the model.
(2) Number of processed frames per second (FPS):
  FPS refers to the processing time on a particular hardware platform.
(3) Millions of parameters:
  The number of parameters directly represent the scale of the network.

#### 4.3.2. Accuracy

We use the mean intersection over union (mIoU) to measure the accuracy of the prediction results, which is the class-averaged ratio of the intersection of the pixel-wise classification results with the ground truth to their union [2].

### 4.4. Qualitative Analysis of Segmentation Results

**Figure 6:**
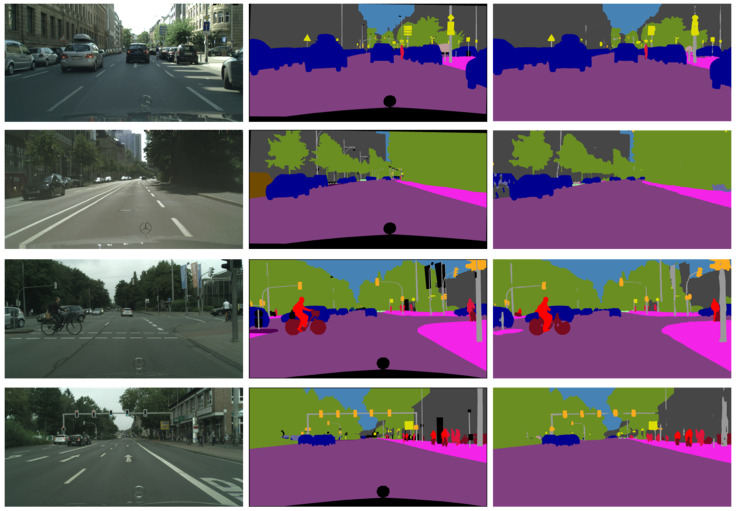
Qualitative results on Cityscapes. First column: Original input images; second column: Labels of ground truth; and Third column: Our segmentation results.

Figure 6 below demonstrates our segmentation results compared with the original input and ground truth results.

**Figure 7:**
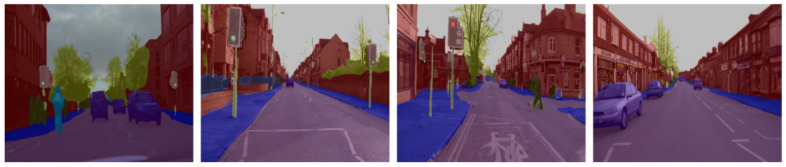
Segmentation results on the Camvid test set.

**Figure 8:**
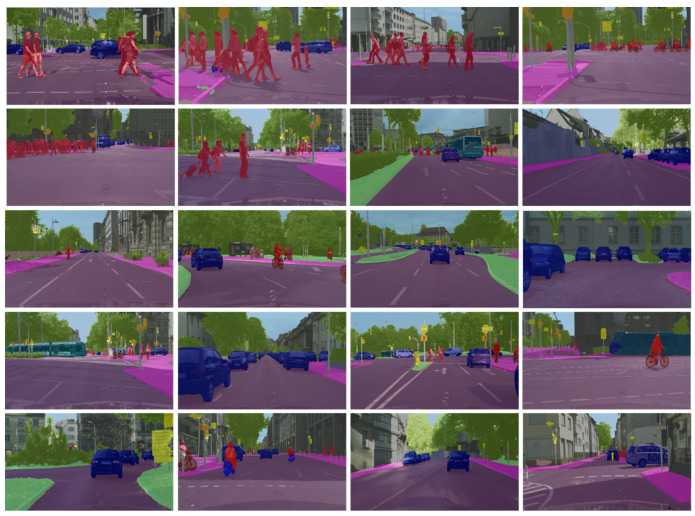
Segmentation results on Cityscapes by HFEN. We mask the prediction results over the original images to visualize the segmentation result.

Figure 7 and Figure 8 below exhibit our segmentation results on Camvid and Cityscapes, respectively. Unlike Figure 6, a mask (a weighted average operation) is placed on the original images to evaluate segmentation accuracy. As can be seen from the figure, the method is robust in complex scenes and is capable of differentiating objects of different sizes (e.g., different sizes of pedestrians and shadows).

### 4.5. Quantitative Analysis of Speed vs Accuracy

#### 4.5.1. Cityscapes

A comparison of comprehensive performances of different methods is listed in Table 2 below. Although experiments are conducted under different conditions (resolution and GPUs), our method achieves competitive results measured by the above-mentioned metrics. For an input size of 2048 × 1024, we have only 5.45 GFLOPs and 1.1 M parameters, which are relatively small. In addition, we also achieve 69.5% mIoU on test set with 112 FPS.

Compared with other models, including PSPNet [16] (412.2 GFOLPs and 250.8 M parameters), DeepLab [21] (457.8 GPOLPs and 262.1 M parameters), and FCN+PPAM+SAM [36] (38.7 GPOLPs and 42.41 M parameters), our model is considerably small.

In terms of accuracy, our model outperforms most of the listed methods, and is only lower than PSPNet [16], which adopts a heavy structure and thus has much slower inference speeds than ours.

Compared with lightweight models, including ENet [37] and ESPNet [38], we exceed them in mIoU by 12.5% and 9.2%, respectively with equivalent speed. Similarly, we also exceed DFANet B [24] in accuracy with comparable GFLOPs and speed. It is worth noting that we process input images with a much higher resolution (2048 × 1024) than them, which means that our model is more efficient.

Compared with ICNet [22] and Fast-SCNN [29], which all process 2048 × 1024 inputs as well, HFEN achieves the same accuracy as ICNet [22], but is more lightweight with much smaller GFLOPS. We also achieve a slightly higher mIoU than Fast-SCNN. Moreover, HFEN is more accurate and efficient than ERFNet [39] and BiSeNet [19] (using Xception39 backbone), with a 1.5% and 1.1% test mIoU gain, respectively.

#### 4.5.2. Camvid

Similar to Table 2, the comparison of performances on Camvid is made among different methods which is shown in Table 3 below. HFEN achieves a 66.1% mIoU on the CamVid test set at 127 FPS with 1.81 GFLOPs, using only CamVid training data for training. It also demonstrates competitive results in segmentation accuracy and inference speed. Our method even outperforms accuracy-oriented method DeepLab [21] by a healthy margin, and also has a big accuracy gain over SegNet [17], DPN [41], SDT [42], and BiSeNet [19]. Finally, we realized a slightly better overall performance over DFANet A [24] with a higher mIoU, and made a comparable score with DFANet B [24].

### 4.6. Ablation Study

An ablation study has been carried out on Cityscapes to test the effectiveness of both the encoder and decoder separately.

#### 4.6.1. Encoder

We analyze the efficiency of our encoder by replacing it with ResNet-50 [18], ResNet-18, MobileNetV2 [32], and Xception [31] (as compared in Table 4 below):

Still, we test a 2048 × 1024 resolution input. For ResNet-50, the network yields 76.5% mIoU at the cost of 28 million parameters and 367GFLOPs. For ResNet-18, the networks gain a 0.8% accuracy with roughly 10 times the parameters of our method. For MobileNetV2 and Xception, our network surpasses them both in speed and accuracy.

Next, we double and halve the width of our encoder to test the corresponding results.

As shown in Table 5 above, the accuracy of our current model is higher than both the half-sized model and double-sized version. It is a common belief that larger models generally perform better than narrower ones. However, as MobileNetV2 [32] analyzed and pointed out that in neural networks there are some redundant channels, while the “manifold of interests” is embedded in lower-dimensional subspaces, which is also the main motivation for a trade-off between computation and accuracy through scaling operations until the “manifold of interests span the entire network”. As for Cityscape, doubling the network width did not exceed our current structure due to such redundancy.

#### 4.6.2. Decoder

The success of our encoder is due in large part to:

(1) The multi-level pyramid pooling module; and
(2) The feature aggregation module based on DSConv layers.

To analyze the effectiveness of our decoder, we tentatively remove the pyramid pooling, which reduces a small amount of GFLOPs and parameter, but it leads to a 3% mIoU loss.

Then we replace the DSConv layer with normal convolutional layers. It turns out that the model expands to 1.42 million parameters and 8.84 GFLOPs, with only a 0.2% mIoU gain.

The corresponding results are shown in Table 6 below:

### 4.7. Testing on Lower Input Resolution

With the popularity of mobile devices, which process lower resolution inputs. A suitable segmentation scheme is required. We therefore evaluate the performance of our method on half, and quarter input resolutions (as shown in Table 7 below).

For quarter resolution, our method achieves 47.8% mIoU with only 0.34 GFLOPs. For half resolution, a competitive 61.8% mIoU with only 1.36 GFLOPs is reached, which is obviously better than ESPNet [38] and ENet [37]. This result reflects a fact that our network is highly compatible to mobile devices with direct applications.

### 4.8. Segmentation Results on Other Datasets

**Figure 9:**
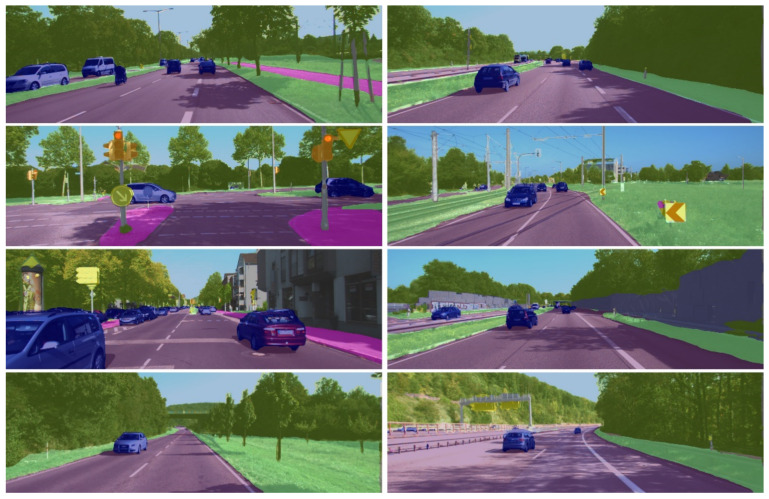
Segmentation results on KITTI dataset.

**Figure 10:**
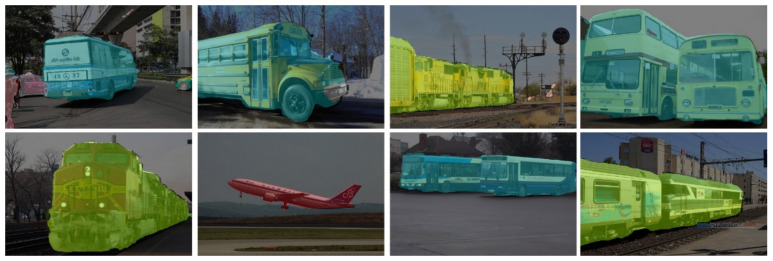
Segmentation results on PASCAL VOC 2012 Dataset.

Apart from Cityscape and Camvid, we display our segmentation results on KITTI [43] and PASCAL VOC 2012 [44] (as shown in Figure 9 and Figure 10 below). KITTI (Karlsruhe Institute of Technology and Toyota Technological Institute) are popular datasets for mobile robotics and autonomous driving. It consists of 200 semantically annotated training samples as well as 200 testing samples. The data format and metrics comply with Cityscapes Dataset. The PASCAL Visual Object Classes (PASCAL VOC) 2012 dataset contains 20 categories including persons, vehicles, planes, boats, buses, cars, trains, etc. Each of them has pixel-level segmentation annotations, which has been widely used as a benchmark for semantic segmentation tasks.

## 5. Conclusions

In this paper, a lightweight architecture is presented (called HFEN) towards fast semantic segmentation of road scenes. The key idea is the encoder-decoder framework with a hierarchical design. Extensive experiments have been conducted on standard benchmarks, in which our network achieved a competitive result without Cityscapes coarse data and extra ImageNet data. It should be noted that most existing networks use advanced backbones. Whereas, we only utilized inverted residual bottleneck modules. Despite its simplicity, our network is efficient, striking a balance between accuracy and inference speed. Ablation studies have been carried out to testify the effectiveness of the encoder and decoder as well as input with different resolutions.

We are also conducting research in autonomous driving, where light detection and ranging (Lidar), inertial devices, and GPS, etc. are all equipped to enhance environmental awareness and navigation. The proposed scheme in this work could be regarded as an imagery sensor of multi-sensor fusion systems used for environmental perception for autonomous driving. It also provides a foundation for deep learning-based monocular depth estimation, which we plan to implement in the near future.

## Figures and Tables

**Table 1:** The structure of our encoder. Ch, n, and s denote the channel number, number of the module, and stride, respectively. exp denotes the expansion ratio, which refers to the ratio of channels between the input and the inner feature maps in the inverted residual block.

Input	Block	exp	Ch	*n*	s
1024 × 2048 × 3	Conv2D	-	32	1	2
512 × 1024 × 32	DSConv	-	48	1	2
256 × 512 × 48	DSConv	-	64	1	2
128 × 256 × 64	bottleneck	6	64	3	2
64 × 128 × 64	bottleneck	6	96	3	2
32 × 64 × 96	bottleneck	6	128	3	1

**Table 2:** Comparative results of accuracy vs speed on Cityscapes, including the results on both validation sets and test sets. We compare our method with other popular networks. We also report the test conditions, including Graphics Processing Unit(GPU) used, Frames Per Second(FPS), Giga floating-point operations per second (GFLOPs), etc.

Model	Resolution	GFLOPs	Params (M)	mIoU (%)		Speed (FPS)	GPU
				Val	Test		
PSPNet [16]	713 × 713	412.2	250.8	-	81.2	0.78	TitanX
BiANet [40]	-	-	-		66.6	-	-
DeepLab [21]	1024 × 512	457.8	262.1	-	63.1	0.25	TitanX
FCN+PPAM+SAM [36]	224 × 224	38.7	42.4		66.5	-	-
SegNet [17]	640 × 360	286	29.5	-	57	16.7	TitanX
ENet [37]	640 × 360	3.8	0.4		57	135.4	TitanX
ICNet [22]	2048 × 1024	270	26.5	-	69.5	30.3	TitanX M
ESPNet [38]	1024 × 512	13	0.4	-	60.3	113	TitanX
ERFNet [39]	1024 × 512	27.7	20	70	68	41.7	TitanX M
Fast-SCNN [29]	2048 × 1024	-	1.1	68.6	68	123.5	TitanX
DFANet B [24]	1024 × 1024	2.1	4.8	-	67.1	120	TitanX
BiSeNet [19]	1536 × 768	14.8	5.8	69	68.4	105.8	GTX 1080Ti
Ours	2048 × 1024	5.45	1.1	71.5	69.5	112	RTX 2080Ti

**Table 3:** Comparison of overall performance on Camvid.

Model	mIoU (%)	Speed (FPS)	GPU
DeepLab [21]	61.6	4.9	TitanX
SegNet [17]	46.4	46	TitanX
ENet [37]	51.3	-	
DPN [41]	60.1	1.2	TitanX
SDT [42]	61.6	-	
BiSeNet [19]	65.6	-	
DFANet A [24]	64.7	120	TitanX
DFANet B [24]	59.3	160	TitanX
Ours	66.1	147	RTX 2080Ti

**Table 4:** Various types of encoders on Cityscapes.

Encoder	mIoU (%)	Params(M)	GFLOPs
ResNet-50 [18]	76.5	28.72	367.44
ResNet-18 [18]	72.3	11.31	89.24
MobileNetV2 [32]	69.8	1.89	67.02
Xception [31]	67.5	1.99	53.67
Ours	71.5	1.1	5.45

**Table 5:** Results of a different width of our networks on Cityscapes.

Encoder	mIoU (%)	GFLOPs
Ours	71.5	5.45
Double	68.1	16.58
Half	62.9	1.71

**Table 6:** Ablation study of modules in the decoder on Cityscapes.

Decoder	mIoU (%)	Params (M)	GFLOPs
Ours	71.5	1.1	5.45
Without ppm	69.7	1.1	5.41
With Conv	71.7	1.4	8.87

**Table 7:** Results of various resolution inputs on the Cityscape validation set.

Resolution	GFLOPs	mIoU (%)
2048 × 1024	5.45	71.5
1024 × 512	1.36	61.8
512 × 256	0.34	47.8

## Data Availability

Not applicable.
